# Impact of C4BPA on Muscle progenitor cell differentiation: insights for Duchenne muscular dystrophy treatment

**DOI:** 10.1038/s41419-026-08588-2

**Published:** 2026-03-18

**Authors:** Esther Fernández-Simón, Ainoa Tejedera-Villafranca, Xiomara Fernández-Garabay, James Clark, Panagiotis Katsikis, Priyanka Mehra, Andrew Galloway, Alexandra Monceau, Elisa Villalobos, Dan Cox, Javier Ramón-Azcón, Juan M. Fernández-Costa, Jordi Díaz-Manera

**Affiliations:** 1 John Walton Muscular Dystrophy Research Centre, Institute of Genetic Medicine, Newcastle University, Newcastle Upon Tyne, UK; 2 Institute for Bioengineering of Catalonia (IBEC), The Barcelona Institute of Science and Technology (BIST), Barcelona, Spain; 3 Institució Catalana de Recerca i Estudis Avançats (ICREA), Passeig de Lluís Companys 23, Barcelona, Spain

**Keywords:** Cell biology, Pathogenesis

## Abstract

Fibroadipogenic precursor cells (FAPs) are key contributors to the fibrotic and adipogenic remodeling observed in Duchenne muscular dystrophy (DMD), yet their precise role in muscle degeneration remains unclear. In this study, we investigated how FAPs from DMD muscle influence myogenesis by conducting co-culture experiments using healthy myoblasts with FAPs derived from either healthy or DMD biopsies, in both direct and indirect contact conditions. We observed that healthy FAPs enhance myogenic differentiation, increasing myotube area, while DMD FAPs significantly inhibit it. To identify underlying molecular mechanisms, we performed mass spectrometry of the FAP secretome and found that 368 of 760 detected proteins were upregulated in DMD FAPs, including C4b-binding protein alpha chain (C4BPA), which showed a notable increase. In vitro treatment of myoblasts with recombinant C4BPA led to severe impairment of myotube formation, evidenced by reduced myotube area, fewer nuclei per myotube, and smaller myotube size. C4BPA exposure also downregulated myogenic markers and upregulated genes associated with muscle atrophy. These findings were further validated in a 3D engineered muscle model, where C4BPA significantly reduced contractile function. Importantly, silencing C4BPA in DMD-derived cultures partially restored myogenic capacity, improving both the differentiation index and nuclear content per myotube. Together, our data demonstrate that DMD FAPs exert anti-myogenic effects, at least in part, through elevated secretion of C4BPA, which interferes with muscle differentiation and function. This highlights C4BPA as a novel effector of muscle degeneration and a promising therapeutic target for modulating the fibrotic environment in DMD. Targeting specific secreted factors from pathological FAPs may help preserve muscle regeneration and mitigate disease progression in dystrophic muscles.

## Introduction

Duchenne muscular dystrophy (DMD) is a genetic disease characterized by progressive degeneration of the skeletal muscle, leading to permanent weakness and disability. Most patients die during the third decade of life due to cardiac or respiratory complications [1]. This debilitating condition arises due to mutations in the dystrophin gene, leading to the reduction or absence of dystrophin protein in muscle fibers [2]. As a result, the skeletal muscle of individuals with DMD undergoes a cascade of pathological changes, including muscle fiber necrosis, inflammation, and excessive fibrofatty tissue deposition [3]. These alterations ultimately compromise muscle function, leading to permanent weakness and disability.

The process of muscle degeneration in DMD involves a complex interplay between different types of cells present in the muscle. Among these cellular players, fibroadipogenic progenitor cells (FAPs) and myoblasts have garnered considerable attention for their potential roles in modulating disease progression [4, 5]. Myoblasts originate from satellite cells, which are responsible for the growth and regeneration of muscle tissue. Myoblasts are the muscle cells that undergo terminal differentiation into myotubes, structures that play a critical role in the regenerative response following muscle injury [6]. In DMD, myoblasts attempt to regenerate muscle fibers in an altered microenvironment characterized by chronic inflammation and extensive fibrosis [7]. FAPs are mesenchymal muscle-resident stem cells characterized by the expression of platelet-derived growth factor receptor alpha (PDGFRα) and have a pivotal role in muscle physiology as they can either promote muscle regeneration or contribute to muscle degeneration by expanding fibrotic and fatty tissue [8, 9]. In healthy muscle, after an acute muscle damage, FAPs proliferate and release components of the ECM to serve as a scaffold where the newly regenerated myofibers will contribute to tissue maintenance and repair [10]. Later in this process, when muscle regeneration is completed, excessive FAPs are cleared by apoptosis mediated by tumour necrosis factor-alpha (TNF-α), returning to normal numbers [11]. However, in DMD, the functional behaviour of FAPs becomes altered, leading to their aberrant activation and excessive fibrofatty infiltration within the muscle tissue [12]. There is growing evidence suggesting that the interactions between different cellular components within the muscle microenvironment play a pivotal role in the pathogenesis of DMD [13]. Although it seems that FAPs have a different behaviour in DMD patients than in healthy muscle [14], the different pathways and pathomechanisms regulating the function of these cells are unknown.

In recent years, considerable research efforts have been directed towards exploring the dynamics of muscle cells and FAP cells in both the context of muscle dystrophies and healthy muscle tissue. Co-culture models have emerged as invaluable tools for dissecting the complex interplay between these cell types. Previous studies in mice have shown that muscle injury stimulates FAPs to maintain tissue homeostasis and support muscle regeneration in healthy muscle by contributing to the ECM remodeling. However, in muscle dystrophies, co-culture studies have shown that the excessive FAP activation impairs the muscle regeneration [9, 15]. Regarding the crosstalk between FAPs and myogenic lineage in human cells, there is a considerable gap in studies performed with human samples. Claudine Moratal et al. analysed the effect of myoblasts on FAPs isolated from skeletal muscles of healthy young and DMD patients. Their results showed that the regulation of FAP differentiation by myotubes was disrupted when myoblasts were derived from DMD patients [16]. However, the role of FAPs in myoblast differentiation has not been addressed yet. Understanding how FAPs influence myoblast behaviour is paramount to elucidating the pathophysiological mechanisms underlying the disease and identifying potential therapeutic targets.

This study aims to analyse the intricate interactions between FAPs and myoblasts in DMD-affected muscle compared to healthy muscle tissue. By examining the crosstalk between these two cell types, we shed light on the molecular mechanisms driving disease progression and explore new potential therapeutic options aimed at restoring muscle function in DMD.

## Materials and methods

### Human skeletal muscle explant culture

Muscle biopsies from healthy controls and from DMD patients were included in the study. Control samples were obtained in collaboration with the Orthopaedic Surgery Department at RVI, and muscle samples of boys with genetically confirmed DMD were obtained for diagnosis purposes or for research from patients seen at the Newcastle Hospital NHS Foundation Trust or at the Hospital Sant Joan de Deu in Barcelona. Clinical information of DMD patients is detailed in Table 1.

**Table 1:** Clinical information of DMD samples collected.

Sample	Age	Sex	Corticosteroids
DMD 1	9	Male	No
DMD 2	10	Male	Yes
DMD 3	7	Male	Yes
DMD 4	8	Male	Yes
DMD 5	10	Male	Yes
DMD 6	9	Male	Yes

### Cell co-culture

Different conditions were created when using direct co-culture: Sorted FAPs were cultured on 24-well culture plates with growth medium. Then, different conditions were created in direct co-cultures: non-differentiated FAPs and adipogenic differentiated FAPs were used. In the non-differentiated FAPs condition, myoblasts were added the next day at a 1:1 ratio into the 48-well plate and, 1 day later, differentiated into myotubes. Myogenic differentiation was induced by using DMEM- GlutaMax with 5% Horse Serum and 1% PS for 7 days. In adipogenic-differentiated condition, FAPs were plated into the 48 well-plate and, 1 day later, differentiated into adipocytes using StemPro adipogenesis differentiation kit (Gibco) for 6 days. Myoblasts were added the next day at a 1:1 ratio into the 48-well plate and, 1 day later, differentiated into myotubes.

For indirect co-culture, cell culture inserts with 1.0-μm pores and 24-well culture plates were used. Healthy myoblasts were plated in the bottom of the 24well-plate and FAPs from healthy or DMD patients were plated into the upper insert. The next day, the transwell co-culture was changed to Skeletal Muscle Cell Differentiation Medium for 7 days.

### In vitro differentiation assays

Human sorted FAPs were used for co-culture experiments. Non-differentiated FAPs were maintained in growth media while adipogenic differentiated FAPs were plated into the 48 well-plate and, 1 day later, differentiated into adipocytes using StemPro adipogenesis differentiation kit (Gibco) for 6 days.

Human immortalized myoblasts were obtained from Institut de Myologie [17]. Myogenic differentiation with immortalized myoblasts or primary myoblasts in co-culture was induced in the same conditions, by culturing confluent cells with DMEM- GlutaMax with 5% Horse Serum and 1% PS. The effect of human C4BPA recombinant protein (Abbexa) in muscle differentiation was evaluated by measuring the fusion index and the area of myotubes after 7 days in differentiation myogenic media. To measure whether C4BPA induced myotube hypotrophy, we added C4BPA (1 μg/mL) at day 0 and day 3, and we compared myotube area using myosin heavy chain staining (clone MF20; Hybridoma bank, IA) with the nontreated condition. For gene expression analysis, cells were harvested on days 0, 3, 6 and 9.

### siRNA

The silencing RNA (siRNA) for C4BPA, as well as the non-targeting control siRNA and transfection reagents, were purchased from Invitrogen. Cells were seeded in 6-well plates at a density of 5000 cells/cm ^2^. FAPs were transfected with C4BPA-siRNA or control siRNA and cultured for 48 h. Cationic lipid complex was prepared by incubating 50 nM siRNA with 3 µl of Lipofectamine® RNAiMAX Transfection reagent (Invitrogen, Carlsbad, CA, USA) in 500 µl of Opti-MEM® I Reduced Serum Medium (Invitrogen) for 15 min and added to the cell medium. After 16 h of incubation, the medium was replaced with fresh medium. Cells were then collected and processed for co-culture conditions. Transfected FAPs were indirectly co-cultured using inserts with 1.0-μm pores and 24-well culture plates. Healthy myoblasts were plated in the bottom of the 24well-plate and transfected FAPs were plated into the upper insert. The next day, the transwell co-culture was changed to Skeletal Muscle Cell Differentiation Medium for 7 days. The C4BPA siRNA, 5’-GCAAGUAGAGAUUAAGACAdTdT-3’ and MISSION® siRNA Universal Negative Control were synthesized by Merck Life Science.

### Statistics

The sample size was determined based on the practical limitations of obtaining primary muscle biopsies from both DMD patients and healthy controls. We focused on maximizing biological relevance and experimental reproducibility by ensuring the technical and biological replicates were sufficient to observe consistent trends across downstream assays. We confirmed that data on cell population did not follow normal distribution using the Shapiro–Wilk test and therefore used nonparametric studies for independent groups to identify significant differences. Statistical analysis of the groups was performed using a one-way ANOVA Test. When ANOVA revealed significant differences, the Tukey post hoc test was performed. A two-way ANOVA followed by Tukey’s post hoc test was used when groups were considered as independent factors. All experiments were performed in triplicate. GraphPad Prism version 10 was used for all statistical tests and plot generation (La Jolla, CA, USA).

## Results

### In vitro model of myoblast and FAPs co-culture

**Fig. 1:**
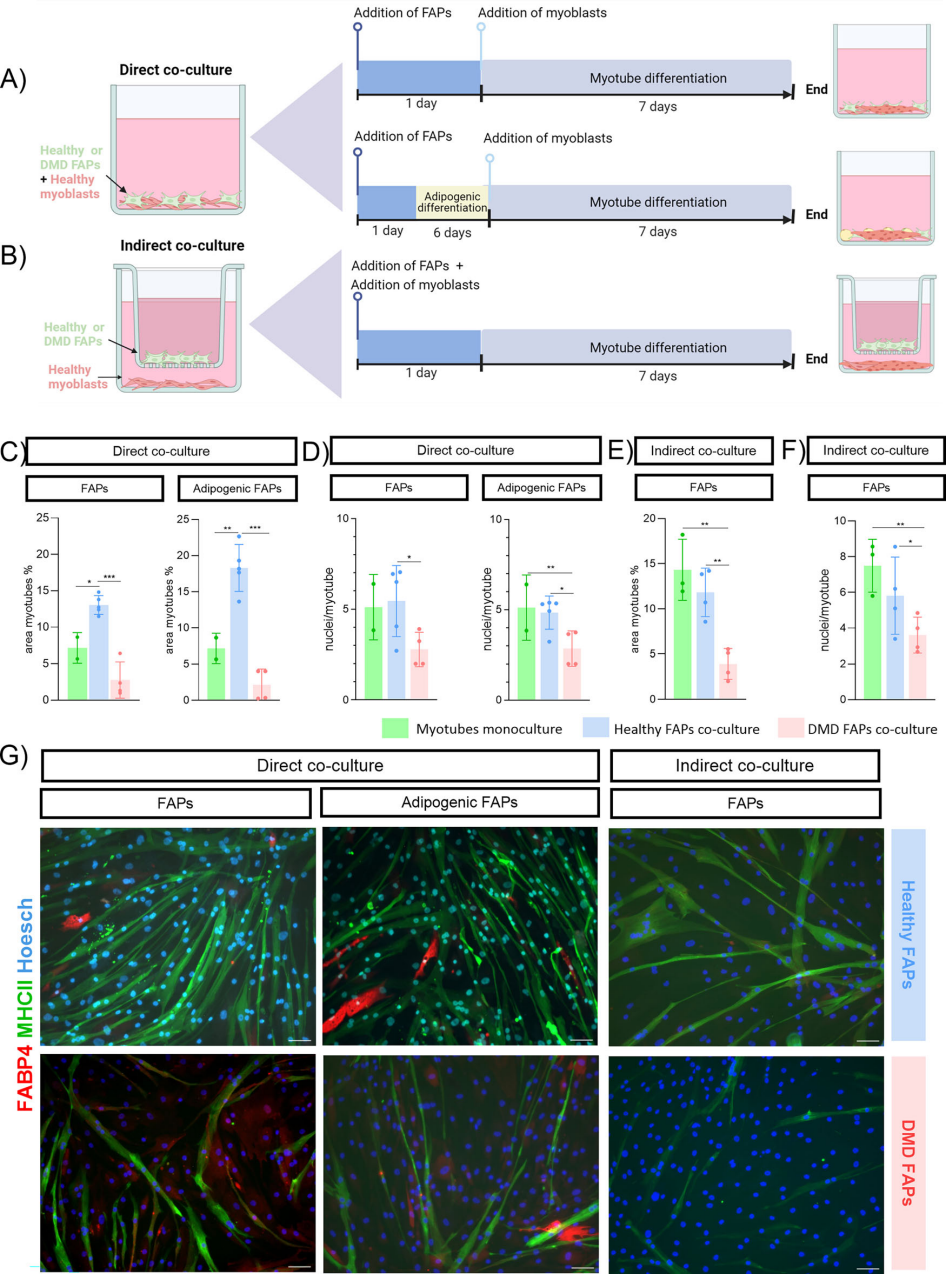
Co-culture system of healthy human myoblasts with healthy and DMD human FAPs. **A** Diagram of myoblast-FAP direct co-culture of undifferentiated FAPs and adipogenic differentiated FAPs. **B** Diagram of myoblast-FAP indirect co-culture of undifferentiated FAPs. **C** Bar graph showing % of myotubes per field measured by MHCII staining, measured at the end of the differentiation process in direct co-culture of non-differentiated FAPs and adipogenic differentiated FAPs. **D** Bar graph nuclei/myotube ratio measured at the end of the differentiation process in direct co-culture of non-differentiated FAPs and adipogenic differentiated FAPs. **E** Bar graph of % of myotubes per field measured by MHCII staining, measured at the end of the differentiation process in indirect co-culture of non-differentiated FAPs. **F** Bar graph of nuclei/myotube ratio measured at the end of the differentiation process in indirect co-culture of non-differentiated FAPs. **G** Representative immunostaining of the different co-culture systems analysed at the end of the differentiation process (day 7). Data are shown as means ± SD; Results were statistically analysed using one-way ANOVA followed by Tukey post hoc test. Statistical significance was set at *P* < 0.05. **P < 0.01; ***P < 0.001.

The role of FAP and satellite cell interactions in human muscle regeneration is not well understood. We co-cultured myoblasts with non-differentiated and adipogenic FAPs from healthy (*n* = 5, 10 ± 1.9 years) and DMD (*n* = 4, 9.75 ± 1.25 years) donors using direct-contact systems (Fig. 1A). Healthy FAPs, regardless of differentiation state, enhanced myotube formation, increasing myotube area. In contrast, DMD-derived FAPs reduced myotube area and nuclei/myotube index under both conditions (Fig. 1C-D). No significant differences were seen between non-differentiated and adipogenic FAPs, suggesting both similarly influence myogenesis, positively in healthy donors, negatively in DMD.

To determine whether DMD FAPs impair myogenesis via direct contact or secreted factors, we used an indirect co-culture system (Fig. 1B). Unlike direct contact, healthy FAPs (*n* = 4, 11.25 ± 0.5 years) did not enhance myotube formation. However, soluble factors from DMD FAPs (*n* = 4, 9.75 ± 1.25 years) significantly reduced myotube area and nuclei/myotube index compared to healthy FAPs (Fig. 1E-G). These findings suggest that direct contact with healthy FAPs supports myogenesis, while DMD FAP-secreted factors impair it.

### Functional analyses of identified secreted proteins

**Fig. 2:**
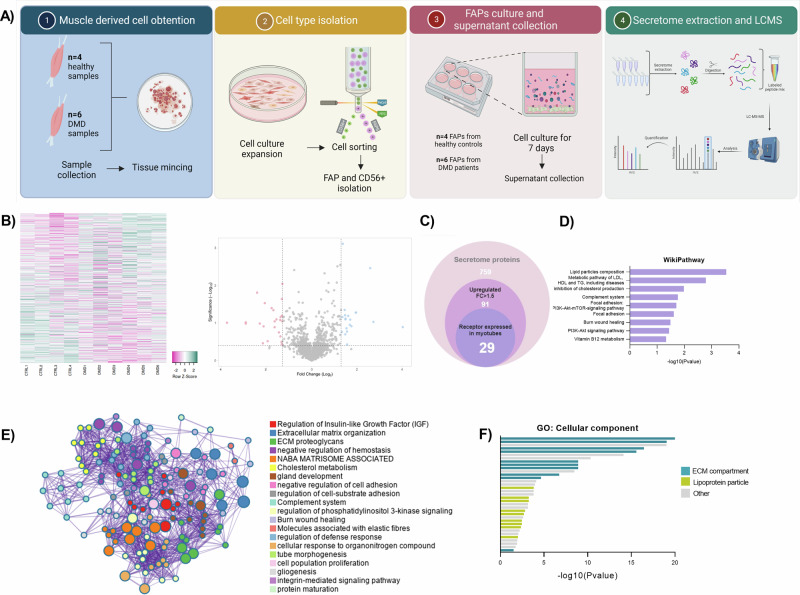
Secretome analysis of DMD isolated from healthy aged-matched controls and DMD patients. **A** Scheme overview of the different steps performed to obtain the secretome data. **B** Heatmap showing the expression values obtained from FAPs from 4 different healthy aged-matched control boys and 6 different DMD patients, and a volcano plot representing the total secretome obtained. **C** Euler diagram showing the number of proteins selected from the secretome analysis. **D** Bar plot showing the enrichment analysis (GSEA) analysed by the WikiPathways database. The X-axis indicated the -log10Pvalue. **E** Network enrichment graph of differentially expressed genes observed in DMD FAPs. **F** Gene ontology analysis of upregulated proteins according to the cellular component.

Building on the observation that DMD FAPs reduce myoblast differentiation by secreted factors, we investigated which factors could be responsible for the observed decreased myotube differentiation. To identify factors secreted by DMD FAPs that inhibit myogenesis, the soluble proteins released by FAPs were analyzed using mass spectrometry. To this end, we cultured FAPs from healthy controls (*n* = 4, mean age ± SD = 8.5 ± 3) and DMD patients (*n* = 6, mean age ± SD = 8.3 ± 1.03) and collected the supernatant after 7 days in culture (Fig. 2A). Among 760 proteins detected, 368 were upregulated in DMD FAPs, with 92 showing >1.5-fold increases. (Fig. 2B). We further filtered our results by selecting proteins that could have a role in myotube formation. Using the datasets published by Deshmukh et al. [18] we obtained the receptors expressed in human myotubes and by using CellTalk DB and Cellinker tools we obtained the ligand-receptor interaction pairs in human samples. This allowed us to narrow our list of proteins to 29 molecules that could have a role in myotube differentiation (Fig. 2C).

The identified secreted proteins were found to be functionally diverse and to be involved in various biological processes. We next characterized differential FAPs secretome from DMD vs healthy control according to biological process and molecular function annotations, and performed statistical enrichment analyses to identify overrepresented categories in our data set. In order to determine the physiological roles of the identified secreted proteins, we performed enrichment analysis (GSEA) of proteins increased in DMD FAPs compared to healthy control FAPs using WickiPathway. WickiPathway term enrichment analysis revealed changes to different categories, including “Lipid particles composition”, “metabolic pathway of LDL” or “Cholesterol production” but also with ECM-related categories, such as “Focal adhesion”, “Wound healing” or “PI3K-Akt signaling pathway” (Fig. 2D). To further understand the molecular mechanisms underlying the disease model Metascape was used to form enrichment networks. The bioinformatic analysis showed that the top enrichment clusters in the DMD-FAPs condition were involved with “Regulation of insulin-growth factor”, “ECM organization”, “ECM proteoglycans” or “Cholesterol metabolism” (Fig. 2E). Finally, the set of altered protein were checked for enrichment of gene ontology (GO) cellular component. Notably, several proteins involved in the ECM compartment and lipoprotein particle were increased (Fig. 2F).

### Identification of FAPs secretome

**Fig. 3:**
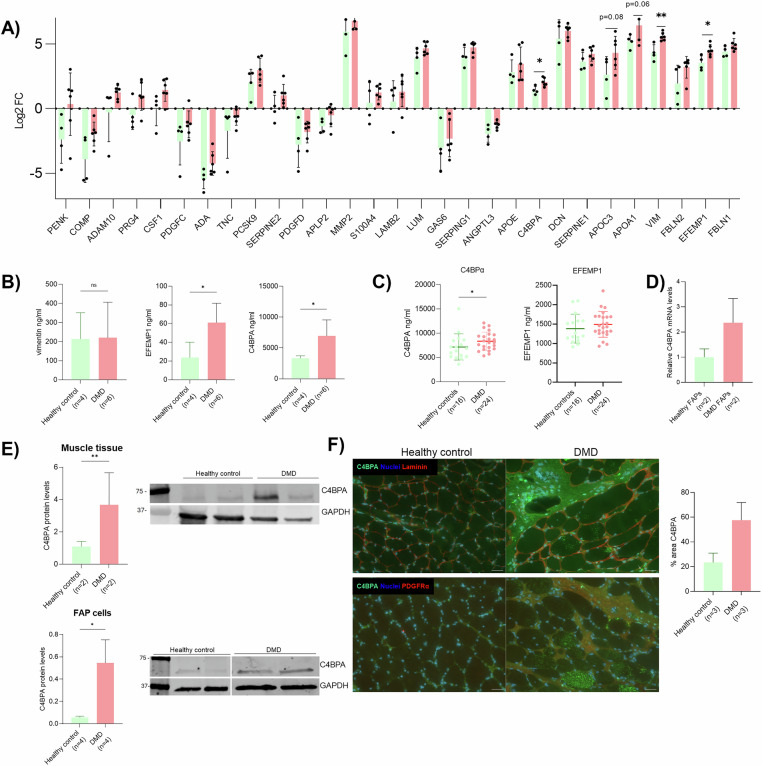
Secretome validation analysis. **A** Bar plot showing fold-change expression of proteins identified in the secretome. **B** Bar plot showing protein levels of vimentin, EFEMP1 and C4BPA in FAPs secretome from healthy aged-matched controls (*n* = 4) and DMD patients (*n* = 6) analysed by ELISA. **C** Dot-plot showing EFEMP1 and C4BPA levels in serological samples from healthy aged-matched controls (*n* = 16) and DMD patients (*n* = 24) analysed by ELISA. Paired t-test was used to analyse. **D** Bar-plot showing the relative mRNA levels of C4BPA analysed in cells (*n* = 2). **E** Western-blot results of C4BPA and GAPDH expression in muscle tissue from healthy controls (*n* = 2) and DMD patients (*n* = 2), and in FAP cells from healthy controls (*n* = 3) and DMD patients (*n* = 4). **F** Representative images of muscle histology from a healthy control young boy and a DMD patient stained with C4BPA, laminin and PDGFRα. Scale bar: 50 µm. and bar plot showing the area of C4BPA expressed in the IF tissue staining from healthy controls (*n* = 3) and DMD patients (*n* = 3). Data are shown as means ± SD; Results were statistically analysed using paired t-test analysis. Statistical significance was set at *P* < 0.05; **P < 0.01; ***P < 0.001.

Using the expression values from the secretome, we further analysed the statistical difference between DMD FAPs and healthy FAPs by using a paired t-test. From the 29 secreted proteins identified, only 3 proteins were statistically different between DMD and control FAPS (Fig. 3A). We further validated these results by analysing the expression of these 3 proteins in the supernatant from healthy and DMD patients by ELISA assay. We observed that EFEMP1 and C4BPA released proteins were increased in the DMD-FAP condition compared to the healthy-FAPs condition. However, vimentin expression was not statistically different (Fig. 3B).

We then focused on the expression of the 2 validated secreted proteins in serological samples from healthy aged-matched controls (*n* = 16, mean age ± SD = 17.3 ± 4.47) and DMD patients (*n* = 24, mean age ± SD = 17.3 ± 3.24). Our results showed that C4BPA serological levels were statistically increased in DMD patients compared to healthy controls. However, EFEMP1 serological levels did not show statistical differences (Fig. 3C). We focused on C4BPA for further characterization. First, we confirmed that C4BPA was more expressed at the mRNA and protein level in FAPs from healthy controls and DMD patients (Fig. 3D, E). Then, we analysed the protein level in muscle tissue from healthy aged-matched controls and DMD patients by Western-blot and immunofluorescence and observed that C4BPA levels were also increased in vivo (Fig. 3E, F).

### Effect of C4BPA on myogenesis

**Fig. 4:**
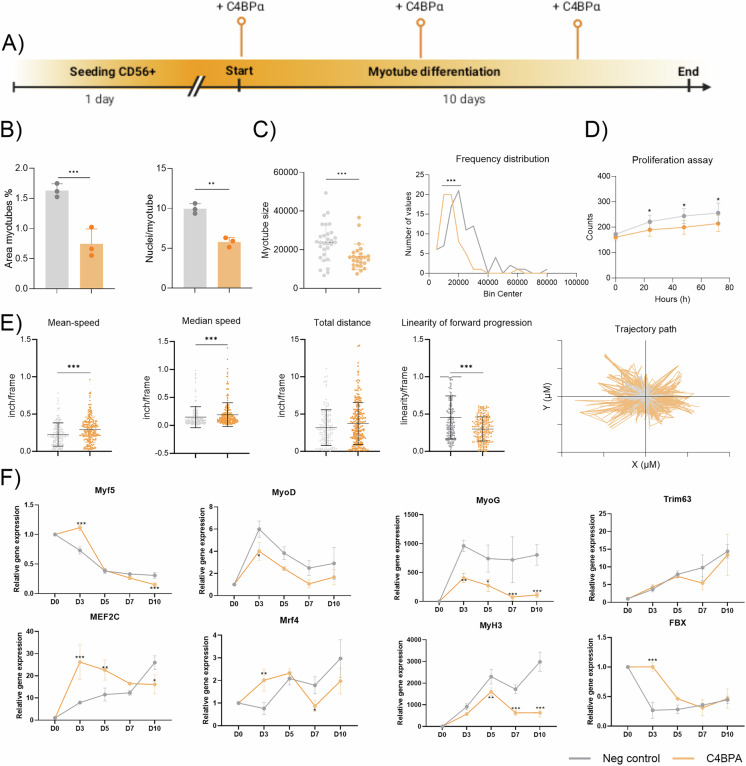
Effect of C4BPA on 2D myogenic differentiation. **A** Timeline scheme of the differentiation of myoblasts with C4BPA treatment. **B** Bar plot showing the % area of myotubes and the ratio of nuclei/myotube of untreated and treated myoblasts with C4BPA after the differentiation process. **C** Myotube size graph and fiber size frequency in untreated and treated myoblasts with C4BPA after the differentiation process. **D** Bar plot showing the proliferation data after 24, 48 and 72 h. **E** Graphs showing different migratory parameters (mean-speed, median speed, total distance, linearity of forward progression and trajectory path of myoblasts after 72 h in culture. **F** Relative expression genes analysed after the differentiation process in non-treated and C4BPA-treated myoblasts. Data are represented as the mean of three replicates ± standard error of the mean. Results were statistically analysed using Mann-Whitney U. Statistical significance was set at *P* < 0.05. **P < 0.01; ***P < 0.001.

Since DMD-FAPs impaired myogenic differentiation and showed elevated C4BPA levels in both supernatant and patient serum, we investigated C4BPA’s role in myogenesis using healthy immortalized myoblasts. A non-toxic dose (1 µg/mL) was chosen after observing toxicity at higher levels (Supplementary Fig. 1B. When added during late differentiation, C4BPA had no significant effect on myotube formation(Supplementary Fig. 1C). However, early exposure significantly reduced myotube area, nuclei/myotube ratio, size, and size distribution (Fig. 4A–C). It also decreased proliferation (48 h, 72 h), increased migration speed, and reduced movement linearity (Fig. 4D, E). These results suggest that early C4BPA exposure impairs myotube formation.

We further assessed gene expression over 9 days. Early C4BPA exposure decreased *MYOD* (day 3), increased *MEF2C* (day 3) but reduced it by day 10, and similarly altered *MRF4*. *MYOG* and *MHC* levels were consistently reduced. Among atrogenes, *FBX* was upregulated at day 3, indicating early atrophic effects (Fig. 4F).

### C4BPA impairs myotube differentiation and muscle contractility in 3D skeletal muscle tissues

**Fig. 5:**
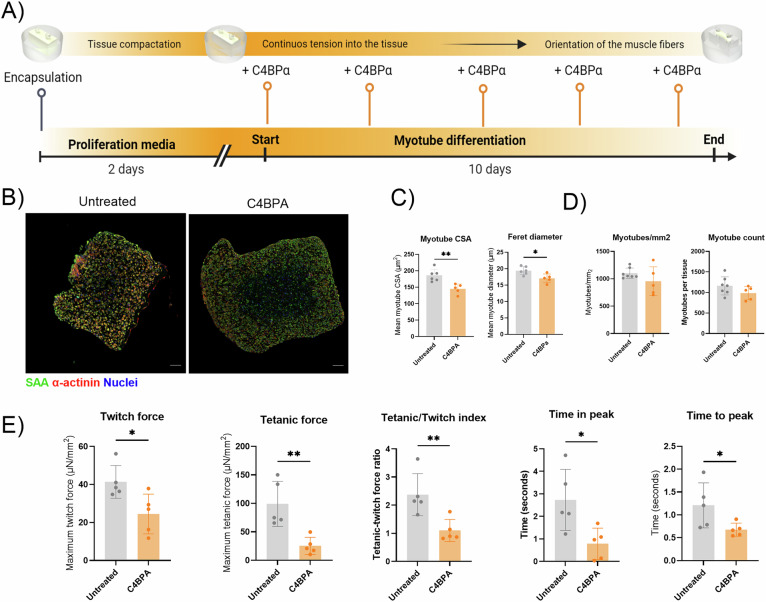
Effect of C4BPA on myotube differentiation in 3D culture. **A** Timeline scheme of the differentiation of the 3D culture with C4BPA treatment. **B** Representative cross-sectional image of a 3D muscle, untreated and treated with C4BPA. Staining corresponds to SAA, F-actinin and nuclei of tissues. **C** Bar graph showing myotube cross-section area (µm2) and Feret diameter (µm) after 10 days myoblasts differentiation with and without C4BPA treatment. **D** Bar graph showing the total number of myotubes measured per mm^2^ or by tissue. **E** Bar graph showing contractile dynamics of the 3D muscle tissue after 10 days of differentiation. Twitch force (µN/mm2), tetanic force (µN/mm2), tetanic/twitch index, time in peak (seconds) and time to peak (seconds) parameters were measured. Data are shown as means ± SD; Statistical significance was set at *P* < 0.05, **P < 0.01.

We used a 3D bioengineered muscle model to assess C4BPA’s impact on muscle function (Fig. 5A). Human myoblasts were embedded in fibrin within PDMS molds to form contractile myotubes responsive to electrical stimulation. C4BPA was applied throughout differentiation. Immunostaining for sarcomeric α-Actinin showed reduced myotube size (cross-sectional area and Feret diameter), with no change in myotube number (Fig. 5B–D). Functionally, C4BPA-treated tissues generated less force during twitch and tetanic contractions, with a lower tetanic/twitch ratio and shortened time to peak and peak duration, indicating impaired contractile function (Fig. 5E).

### RNA silencing of C4BPA in DMD FAPs

**Fig. 6:**
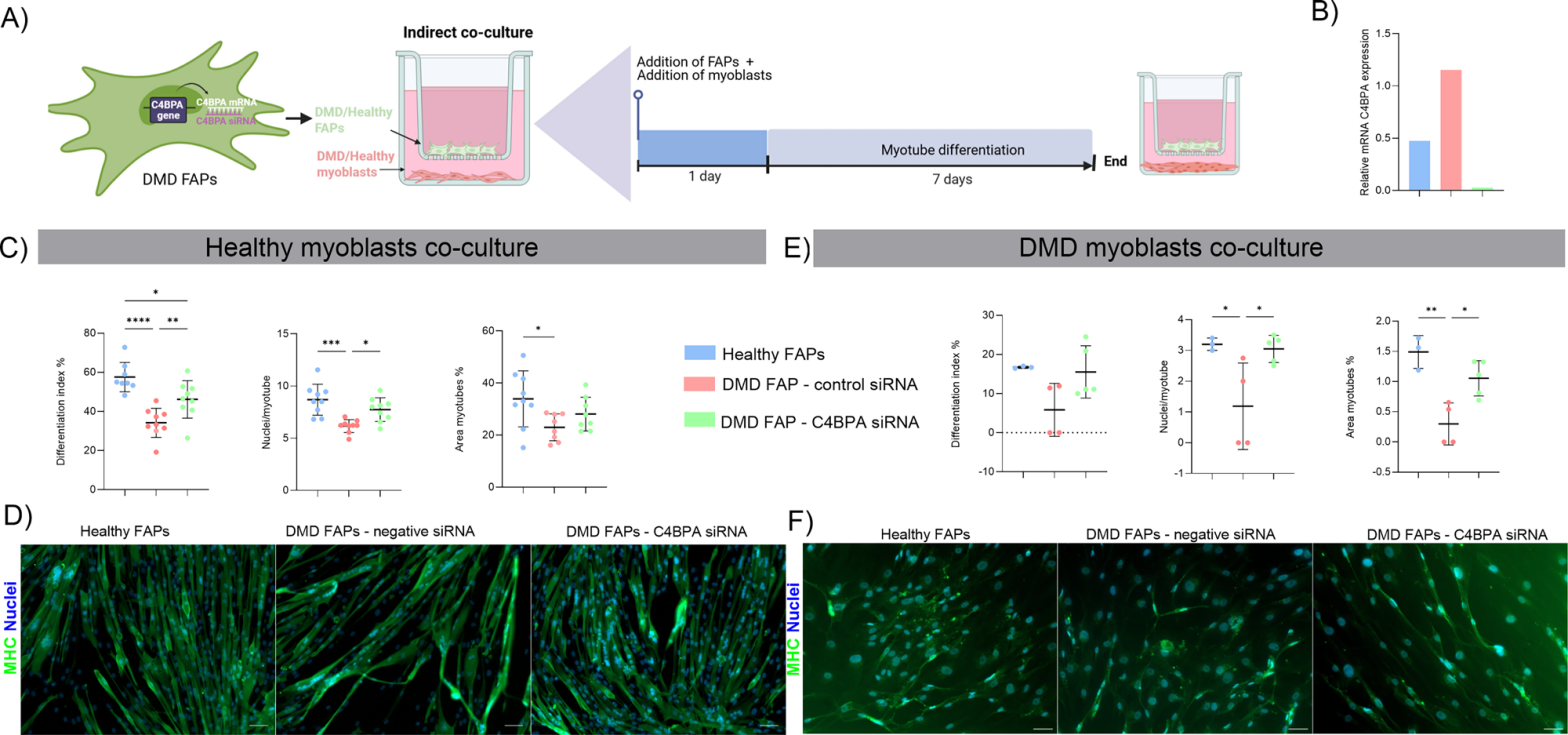
Effect of silencing C4BPA in myotube differentiation. **A** Diagram scheme of the indirect co-culture of C4BPA siRNA-transfected FAPs with healthy myoblasts. **B** Relative expression of C4BPA mRNA levels after transfection (*n* = 1). **C** Dot plot showing the differentiation index of myoblasts after being co-cultured with transfected and non-transfected DMD FAPs and healthy FAPs. **D** Dot plot showing the number of nuclei per myotube of myoblasts after being co-cultured with transfected and non-transfected DMD FAPs and healthy FAPs. **E** % of myotubes per field measured by MHCII staining after being co-cultured with transfected and non-transfected DMD FAPs and healthy FAPs. **F** Representative immunostaining of the indirect co-culture system analysed at the end of the differentiation process after FAPs transfection. Data are shown as means ± SD; Statistical significance was set at *P* < 0.05. **P < 0.01; ***P < 0.001.

To assess the impact of C4BPA inhibition on myogenesis, DMD FAPs were transfected with siC4BPA or control siRNA and co-cultured with healthy myoblasts (Fig. 6A). Knockdown of C4BPA significantly increased the differentiation index and the nuclei/myotube ratio compared to control siRNA (Fig. 6B–D). Although the myotube area was larger in the siC4BPA group, the difference was not statistically significant (Fig. 6E, F). Overall, our results show that silencing of C4BPA in DMD FAPs resulted in a better myotube differentiation compared to non-silenced FAPs.

## Discussion

This study investigated the role of FAPs in myogenesis in DMD patients versus controls. We found that soluble factors secreted by DMD FAPs impair myotube formation, partly through the expression of C4BPA. Silencing C4BPA via siRNA partially restored myogenesis. While skeletal muscle regeneration depends on satellite cells, the contribution of FAPs—extensively studied in mice [16]—remains poorly characterized in humans. Using human co-culture models, we showed that healthy FAPs, whether undifferentiated or adipogenic, promote myogenesis, aligning with previous rodent studies.

We showed that FAPs from healthy young boys positively regulate myogenesis in vitro, either when they are undifferentiated or when they have already differentiated to adipocytes. Conversely, DMD FAPs reduced myotube formation both in direct and indirect co-cultures, suggesting secreted factors drive the impairment. This aligns with murine data showing dystrophic FAPs inhibit myoblast function and promote fibrosis [19, 20]. Our secretome analysis revealed that DMD FAPs release proteins linked to ECM remodeling and adipogenesis regulation. These changes may reflect and reinforce the altered ECM environment typical of dystrophic muscle [21, 22]. Since ECM both influences and responds to cellular signaling, the disrupted secretome of DMD FAPs likely contributes to impaired regeneration. Whether these differences stem from microenvironmental cues or dystrophin deficiency remains to be determined.

Mechanistically, we showed that C4BPA was increased not only in DMD FAPs but also in serological samples from DMD patients. C4BPA belongs to the complement system family of proteins, which are involved in detecting and removing pathogens, but also regulate cell physiology of immune and non-immune cells [23]. The involvement of the complement system in muscular dystrophies has been addressed in Duchenne muscular dystrophy. Indeed, Engel al. et al demonstrated deposition of C3 and C9 on necrotic fibers [24]. Recently, Florio et al. studied the role of the C1 complement complex on DMD fibrogenesis. They observed that FAPs and macrophages secrete distinct subunits of the C1 complement complex, resulting in activation of the WNT signaling, which was followed by an increased fibrosis in the muscle. However, the authors did not find implications for myoblasts after pharmacological inhibition of C1 [25].

In our study, we have observed that DMD FAPs release an increased concentration of C4BPA protein. Although it is usually expressed as part of the extracellular complement regulator C4b-binding protein (C4BP), it has also been found expressed intracellularly in cancer cells, interacting with NF-Kβ pathway and mediating anti-apoptotic responses [26]. C4BP has various isoforms, and C4BPA is generally expressed at higher levels during inflammation [27]. The C4BPA protein possesses binding sites for heparin, C-reactive protein, CD40, C3b and low-density lipoprotein receptor-related protein 1 (LRP1) [28]. Although only a few studies have addressed the role of C4BPA, the potential use of C4BPA as a biomarker in DMD was suggested by Signorelli et al. The authors observed that C4BPA was associated with disease progression and could discriminate between ambulant and non-ambulant patients in a cohort of 78 patients, being increased in non-ambulant patients compared to ambulant [29].

Here, we found that C4BPA impairs myotube formation by deregulating myogenic transcription factors. Specifically, *MYOD, MYOG* and *MYH3* were downregulated after C4BPA treatment. The implication of lower *MYOD* expression by C4BPA can be linked to the decreased rate of myoblast proliferation that we observed after 48 and 72 h of C4BPA treatment. On the other hand, the lower myogenin and *MYH3* in the late differentiation process of myotube was also evident in the reduced fusion and differentiation index. Moreover, we also found the myocyte enhancer factor 2C (*MEF2C*) to be significantly increased during the differentiation process, except at day 10. *MEF2C* is a transcription factor that regulates skeletal muscle differentiation and growth [30], and when it is constitutively active, it can promote muscle hypertrophy [31]. Indeed, Suarez et al. recently observed that *MEF2C* is one of the transcription factors increased in DMD muscle fibers compared to healthy controls [32]. We suggest that C4BPA might be one of the molecules upregulating *MEF2C* in DMD fibers. Regarding atrophy genes, we observed that C4BPA promoted the upregulation of FBX gene in early myotube differentiation, suggesting an added atrophy-induced mechanism. The impairment of myogenesis after C4BPA treatment was also evident using complex 3D skeletal muscle cell culture methods, which enabled the evaluation of its effect on muscle function. We found that the decrease in myotube size was accompanied by a reduction of contractile forces, as well as evidence of muscle fatigue and lower muscle fiber maturation. These observations suggest that the increased levels of C4BPA found in DMD are affecting the early and late differentiation of the myotube process. The effect of C4BPA on myoblasts was also evident when assessing different migratory parameters. We observed that C4BPA increased the speed of the myoblast but without affecting the total distance and impairing the forward-directed migratory progression. The directional migration of myogenic progenitors is important during the regeneration process of myofibers since, after an acute injury, myoblasts are activated, proliferate and migrate towards sites of injury above the basal lamina [33]. The lack of a forward-directed progression observed by C4BPA could lead to a reduced regenerative response. The characterization of C4BPA as a factor impairing myogenesis was also corroborated by silencing the gene in vitro. We observed that after C4BPA silencing in a human DMD co-culture model with myoblasts, we partially reversed the healthy myogenic phenotype in vitro. Based on these results, we propose that pharmacological blockade of C4BPA could be explored as a therapeutic option for promoting the regeneration process in muscle dystrophies. Since regeneration is impaired in DMD patients, therapies focused on targeting the progression of the disease will be useful to preserve the muscle structure and, consequently, will make patients more receptive to gene therapies, optimizing their effectiveness and delivery.

One of the limitations of this study is the lack of characterization of the effect of C4BPA on FAPs. The muscle environment is a complex tissue that involves different crosstalk of cells. We believe that C4BPA might have a role in other non-myogenic cells, such as FAPs or inflammatory cells. Indeed, studies performed on cows found that C4BPA4 also regulates lipid metabolism, involving the synthesis of triglycerides, total cholesterol and free fatty acids [28]. Further studies unraveling the role of C4BPA on disease progression and on FAPs might elucidate a novel pathway involved in fibroadipogenic expansion in muscle dystrophies.

Overall, we found that DMD FAPs might have a different effect on myoblasts than healthy FAPs since DMD FAPs impair myotube formation. Our work provides characterization of the FAPs secretome and identifies C4BPA as one of the proteins impairing myogenesis. Elucidating the different phenotypes in FAPs in muscular dystrophies is essential to understanding the changes in ECM composition that occur during disease progression. Finding these dysregulations is important for developing targeted therapeutic approaches aimed at stabilizing the ECM and preserving muscle function in affected individuals. Our findings thus provide new insight into mechanisms of impaired regeneration processes in muscle dystrophies as well as novel targets that could be further explored as a therapeutic approach.

## Supplementary information

Supplemental material information

Supplemental Figure 1

Full WB

Cheklist

## Data Availability

All data will be available to readers upon request.
