# Correction: Impact of C4BPA on Muscle progenitor cell differentiation: insights for Duchenne muscular dystrophy treatment

**DOI:** 10.1038/s41419-026-08843-6

**Published:** 2026-05-18

**Authors:** Esther Fernández-Simón, Ainoa Tejedera-Villafranca, Xiomara Fernández-Garabay, James Clark, Panagiotis Katsikis, Priyanka Mehra, Andrew Galloway, Alexandra Monceau, Elisa Villalobos, Dan Cox, Javier Ramón-Azcón, Juan M. Fernández-Costa, Jordi Díaz-Manera

**Affiliations:** 1 John Walton Muscular Dystrophy Research Centre, Institute of Genetic Medicine, Newcastle University, Newcastle Upon Tyne, UK; 2 Institute for Bioengineering of Catalonia (IBEC), The Barcelona Institute of Science and Technology (BIST), Barcelona, Spain; 3 Institució Catalana de Recerca i Estudis Avançats (ICREA), Passeig de Lluís Companys 23, Barcelona, Spain

**Keywords:** Cell biology, Pathogenesis

Correction to: *Cell Death & Disease* 10.1038/s41419-026-08588-2, published online 18 March 2026

In this article the author name Panagiotis Katsikis is incorrectly published as Panogiotis Katsikis.

The original article has been corrected.

