# Supplementary material for: Impact of C4BPA on Muscle progenitor cell differentiation: insights for Duchenne muscular dystrophy treatment
Source: Cell Death Dis. 2026 Mar 18;17(1):313. doi: 10.1038/s41419-026-08588-2 (PMC13039365; doi:10.1038/s41419-026-08588-2)
Supplement: Supplementary file 1 — Supplemental material information [file 41419_2026_8588_MOESM1_ESM.docx]

**Supplemental material**

Cell isolation

FAPs were isolated from healthy and DMD muscle explants and myoblasts from healthy donor explants. PMuscle biopsies were mashed and minced into 2- 3 mm fragments in Hanks’ balanced salt solution with 1% penicillin– streptomycin (PS) (Lonza, Basilea, Switzerland). Muscle fragments were placed in a dish with conditioned media containing M199 (Lonza), 35% fetal bovine serum (FBS) (ThermoFisher [Gibco], Waltham, MA), and 1% PS (Lonza) and incubated for 24 hours at 37℃ and 5% CO2 [17]. Then, growth medium consisting of DMEM- GlutaMAX (Gibco), with 20% FBS (Gibco), 2.5 ng/mL of basic fibroblast growth factor (bFGF) and 1% penicillin– streptomycin (PS) (Lonza, Basilea, Switzerland) was added until cover the muscle explants in the dish. After 5- 7 days, cells started to sprout from muscle explants and these were trypsinized, washed, and stained with the following antibodies for 30 minutes at 4℃: Anti-PDGFRα- biotin (Cat. BAF322, R&D Systems, Minneapolis, MN) and CD56- FITC (Cat. 130- 114- 549). Stained cells were sorted on a FACSAria using FACSDiva software (Becton Dickinson, Ashland, OR). Compensations were adjusted according to the single stained controls. PDGFRα+/CD56− fraction was defined as FAPs and the PDGFRα−/CD56+ fraction as myoblasts. Purified FAPs were subcultured with growth medium consisting of DMEM- GlutaMAX (Gibco), with 20% FBS (Gibco), 2.5 ng/mL of basic fibroblast growth factor (bFGF) and 1% penicillin– streptomycin (PS) (Lonza, Basilea, Switzerland). Purified myoblasts were subcultured with Skeletal Muscle Cell Growth Medium (PromoCell, Heidelberg, Germany).

Histology and Immunofluorescence

To analyse the cell differentiation, expression of FABP4 (Cat. AF3150; 1:100; R&D systems) and myosin-heavy chain (MF-20) in cell co-cultures were used. Plates were fixed with 4% PFA for 10 minutes and blocked with Ultracruz blocking solution (Santa Cruz Biotechnology, Dallas, TX). Primary antibodies were incubated for 1 hour and after washing steps, secondary antibodies were used for 1 hour. Donkey anti-goat Alexa 594 (1:500; ThermoFisher) was used for FABP4 and donkey anti-mouse 488 (1:500; ThermoFisher) was used against MF-20. Nuclei were stained with Hoechst 33342 (Invitrogen, Thermo-Fisher Scientific). Pictures of random fields and of replicates were obtained by using a ZeissAxioimager. Quantitative analysis was performed using ImageJ software (National Institute of Health). Myotube differentiation ratio was analysed by measuring the area of myotubes and the ratio of nuclei/myotube. Area of myotube was quantified by selecting the positive area of each myotube stained by the MF-20 antibody. Then, total nuclei in each myotube were counted using the Hoechst staining. A ratio of nuclei/myotube was represented in a bar-graph.

Immunofluorescence in muscle tissue was performed by using frozen muscle sections obtained with a cryostat (Leica Microsystems, Wetzlar, Germany), fixed with acetone, and incubated with Ultra-Cruz Blocking Reagent (Santa Cruz Biotechnology, Dallas, TX). Muscle tissue was stained using a rabbit anti-C4BPA (AB20044, Antibodies.com), rat anti–laminin-α2 (4H8-2, Enzo), and goat anti-PDGFRα+ (AF1062-SP, R&D system) overnight at 4C. Then, donkey anti-rabbit secondary antibody Alexa Fluor 488 (ThermoFisher), donkey anti-rat 594 antibody Alexa Fluor 594 (ThermoFisher) or donkey anti-rat Alexa Fluor 555 (ThermoFisher) were used as a secondary antibody for 1 h at RT. Following the incubation with the secondary antibody, nuclei were stained with Hoechst 3342 (Invitrogen, Thermo-Fisher Scientific). Images were acquired using a Zeiss AxioImager Apotome fluorescent microscope and analysed using Fiji software. ImageJ software was used to quantify the area of positive staining in muscle tissue. A minimum of five independent fields per staining were quantified.

Western blot

Cell pellets corresponding to each condition were lysed in RIPA buffer (Sigma-Aldrich) containing a protease and phosphatase inhibitor cocktail (Roche, Basel, Switzerland). Lysates were centrifuged at 4 °C at 13000 x g for 20 min and supernatants were stored at − 80 °C. Protein concentrations were determined using Pierce™ BCA Protein Assay (Thermo Fisher Scientific, Waltham, MA, USA). Thirty micrograms of protein were resolved in a 10% sodium dodecyl sulfate (SDS) polyacrylamide gel and transferred to nitrocellulose membranes. Unspecific binding sites were blocked by incubation for 1 h in casein diluted 1:1 in tris-buffered saline (TBS). Blots were incubated overnight with the primary rabbit ani- C4BPA (AB20044, Antibodies.com) and mouse anti-GAPDH (1E6D9, ProteinTech). Secondary antibodies used in the study were GAR700 and GAM700 IR-Dye (Li-Cor, Lincoln, Nebraska, USA). The specific bands corresponding to the proteins of interest were visualized with the Odyssey infrared detection system (Li-Cor) together with the Image Studio software (Li-Cor).

ELISA

Supernatant samples were collected from healthy and DMD-FAPs in cell culture for 6 days. Serological samples were obtained from both healthy and DMD patients. Blood samples were collected and centrifuged for 1600 g for 9 minutes at 4 °C in order to separate the serum. The serum was aliquoted and stored at −80 °C until analysis.

Serum complement 4 binding protein alpha (C4BPA) (Antibodies.com), Vimentin, and Fibulin-3 (EFEMP1) (abcam) levels were measured using commercial enzyme-linked immunosorbent assay (ELISA) kits, according to the manufacturer’s instructions. Samples were measured in duplicate and read on a microplate reader Varioskan™ LUX multimode microplate reader (ThermoFisher).

Cell viability

The effect of increasing concentrations of C4BPA on viability was measured with the PrestoBlue reagent (Invitrogen) following manufacturer’s instructions. Fluorescence was measured at wave- lengths 570 nm excitation and 600 nm emission using a microplate reader (Varioskan).

Proliferation assay

Immortalized myoblasts were plated at a density of 4500 cells/cm^2^ and treated with 1ug/ml of C4BPA. Cell proliferation was carried out using Incucyte live cell imaging system (Essen BioScience, Ann Arbor, MI). Images were taken every 24 h for 3 days. The cell proliferation is expressed as counts per image.

Migration assay

The migration assay was performed using 96-well plates with inserts for 72 hours. Cells were plated in 96-well plates at 1500 cells/cm^2^ and treated with 25 µg/ml of mitomycin C (Sigma-Aldrich, San Luis, MO, USA) for 1 hour. After that, EGF and EFEMP1 was added to the wells in DMEM containing 2%FBS and migration was evaluated using the IncuCyte® system. Images were taken every 4hours and analysed using the Fiji image processing Trackmate package.

Real-time PCR

We performed gene expression assays using the Taqman Fast Advanced Cells- to- CT kit (ThermoFisher) following the manufacturer’s instructions. Briefly, cells were washed with PBS and incubated with lysis solution for 5 minutes. Sample lysate was mixed with the RT Master mix and the reaction to obtain cDNA. qPCR was performed by using the Fast Advanced Master Mix and the QuantStudio™ 7 Flex Real-Time PCR System (ThermoFisher). The mRNA- specific probes were: TRIM63 (Hs00822397_m1), FBXO32 (Hs01041408_m1), MEF2C (Hs00231149_m1), PAX7 (Hs00242962_m1), MyoD1 (Hs00159528_m1), MYOG (Hs01072232_m1), FBXO32 (Hs01041408_m1), C4BPA (Hs00426339_m1) and GAPDH (Hs99999905_m1) (ThermoFisher). Relative quantification was performed using the comparative Ct method.

*In vitro* functional 3D muscle differentiation assays

Human 3D skeletal muscle tissues were fabricated as previously described [1–4]. Briefly, casting molds made of polydimethylsiloxane (PDMS; Sylgard 184 silicone elastomer kit, Dow Corning) were fabricated, consisting of a rectangular-shaped pool, where the cells/hydrogel solution was seeded, and two vertical, flexible PDMS pillars. After cleaning and overnight treatment with 2% Plurionic® F-127 (Sigma-Aldrich) solution in cold PBS (100 μL/well) at 4 °C, human immortalized myoblasts were encapsulated at 2.5 ×10^7^ cells/mL in a cell/hydrogel mixture and polymerized for 30 min at 37 °C. Hydrogel consisted of 30% v/v Corning® Matrigel® Growth Factor Reduced (GFR) Basement Membrane Matrix (Corning®), 2 units/mL of thrombin from human plasma (Sigma-Aldrich) and 4 mg/mL of fibrinogen from human plasma (50% v/v) (Sigma-Aldrich). They were kept in growth media containing 1 mg/mL of 6-aminocaproic acid (ACA) (Sigma-Aldrich) (skeletal muscle basal medium (PromoCell GmbH), skeletal muscle supplemental mix (PromoCell GmbH), 10% Fetal Bovine Serum (FBS) (Gibco™), 100 U/mL penicillin and 100 μg/mL streptomycin (Gibco™)).

To evaluate the effect of human C4BPA recombinant protein in the 3D skeletal muscle tissues, after two days, they were switched to differentiation medium containing C4BPA (0.5 μg/mL) for ten days (Dulbecco's Modified Eagle Medium, high glucose, GlutaMAX™ Supplement (Gibco™), containing 1% KnockOut™ Serum replacement (Gibco™), 1% Insulin-Transferrin-Selenium-Ethanolamine (Gibco™), 1% Penicillin-Streptomycin-Glutamine (Gibco™)) and 1 mg/mL of ACA). Half of the volume of differentiation medium was replaced every two days.

Electrical Pulse Stimulation (EPS) and force measurement

To induce muscle contraction, 3D skeletal muscle tissues were electrically stimulated after twelve days of differentiation. PDMS casting molds were placed on a 24-well plate with fresh differentiation medium and they were settled on a Zeiss Axio Observer Z1/7 outfitted with the XL S1 cell incubator at 37 °C and 5% CO2. A custom-made device consisting of two graphite electrodes per well located on the lid was used to perform the EPS. The device was connected to a pulse generator (Multifunction Generator WF1974, NF Corporation), and tissues were stimulated with electrical square-wave pulses of 1 V/mm, 1 ms of pulse width, and frequencies varying from 1 to 50 Hz. Top-view brightfield videos of the pillars of each sample were taken during EPS to measure the force that the 3D skeletal muscle tissue produced against the pillars when they contracted, and the location of each tissue on the y-axis of the pillar was estimated during the assay using brightfield imaging of the sample. Videos were processed as previously described [5] and contractile forces were normalized by the cross-sectional area of each tissue.

Cryosectioning, immunohistochemistry, imaging and image analysis of 3D skeletal muscle tissues

Human 3D skeletal muscle tissues were fixed in 10% formalin solution (Sigma-Aldrich) for 30 min at room temperature, washed three times with PBS for 5 min, and incubated on 30% sucrose solution in PBS for 48 h at 4 ºC in agitation. Samples were embedded in optimal cutting temperature compound (OCT compound) (PolyFreeze, Sigma-Aldrich) within a disposable plastic Cryomold® (VWR) using a batch of isopentane chilled by liquid nitrogen. They were sectioned with a cryostat (Leica CM1900) to obtain transverse sections at a thickness of 20 μm that were placed on SuperFrost Plus™ Adhesion slides (Fisher Scientific).

Slides containing tissue sections were encircled with a PAP pen (ImmEdge™, Vector laboratories), and sections were permeabilized with PBS-T (0.1% Triton-X (Sigma-Aldrich) in PBS) for 10 min, followed by incubation with blocking buffer (UltraCruz® Blocking Reagent (Santa Cruz Biotechnology) for 30 min at room temperature. Next, samples were incubated with the primary antibody (Monoclonal mouse Anti-α-Actinin (Sarcomeric, SAA) primary antibody (1:200, A7811, Merck Life Science) or monoclonal mouse anti-dystrophin primary antibody (MANDYS01 and MANDYS106) (1:50 each, Wolfson Centre for Inherited Neuromuscular Disease (CIND)) in blocking buffer at 4 ºC overnight. After 3 PBS-T washes of 5 min, the samples were incubated for 45 min at room temperature with Alexa Fluor™ 488-conjugated secondary antibody (polyclonal donkey anti-mouse IgG, 1:200, A21202, Invitrogen) and Alexa Fluor™ 594 Phalloidin (1:400, A12381, Invitrogen). Samples were washed for 5 min with PBS and they were mounted with VECTASHIELD Plus Mounting Medium with DAPI (Palex). Transparent enamel was used to seal the edges of coverslips.

Fluorescence images were taken with a ZEISS LSM800 confocal laser scanning microscope. Images were analysed using the Fiji image processing package. For myotube diameter measurements, segmentation was performed using SAA signal with Cellpose 2 software and ImageJ plugin LabelsToRois to measure myotube area and Feret diameter [6,7].

**Supplemental figures**

**Supplemental figure 1.** A) Representative staining of monoculture using healthy myoblasts used for the co-culture experiments. B) Bar plot representing the viability of myoblast after treatment with different concentrations of C4BPA. Results were statistically analysed using one-way ANOVA followed by Tukey post hoc test. C) Timeline scheme of the differentiation of myoblasts with C4BPA treatment. D) Bar plot showing the % of myotubes per field measured by MHCII and the ratio of nuclei/ myotube of untreated and treated myoblasts with C4BPA after differentiation process. E) Graphs showing the mean of myotube size and the frequency of distribution by size in untreated and treated myoblasts with C4BPA after differentiation process. Results were statistically analysed using Mann-Whitney U. F) Bar-graph and representative WB images showing the relative expression of C4BPA protein levels after transfection of DMD cells (n=1). Results were statistically analysed using one-way ANOVA followed by Tukey post hoc test. Data are shown as means ± SD; Statistical significance was set at P < 0.05. **P < 0.01; ***P < 0.001
