# Supplementary figures and images for: Impact of C4BPA on Muscle progenitor cell differentiation: insights for Duchenne muscular dystrophy treatment
Source: Cell Death Dis. 2026 Mar 18;17(1):313. doi: 10.1038/s41419-026-08588-2 (PMC13039365; doi:10.1038/s41419-026-08588-2)

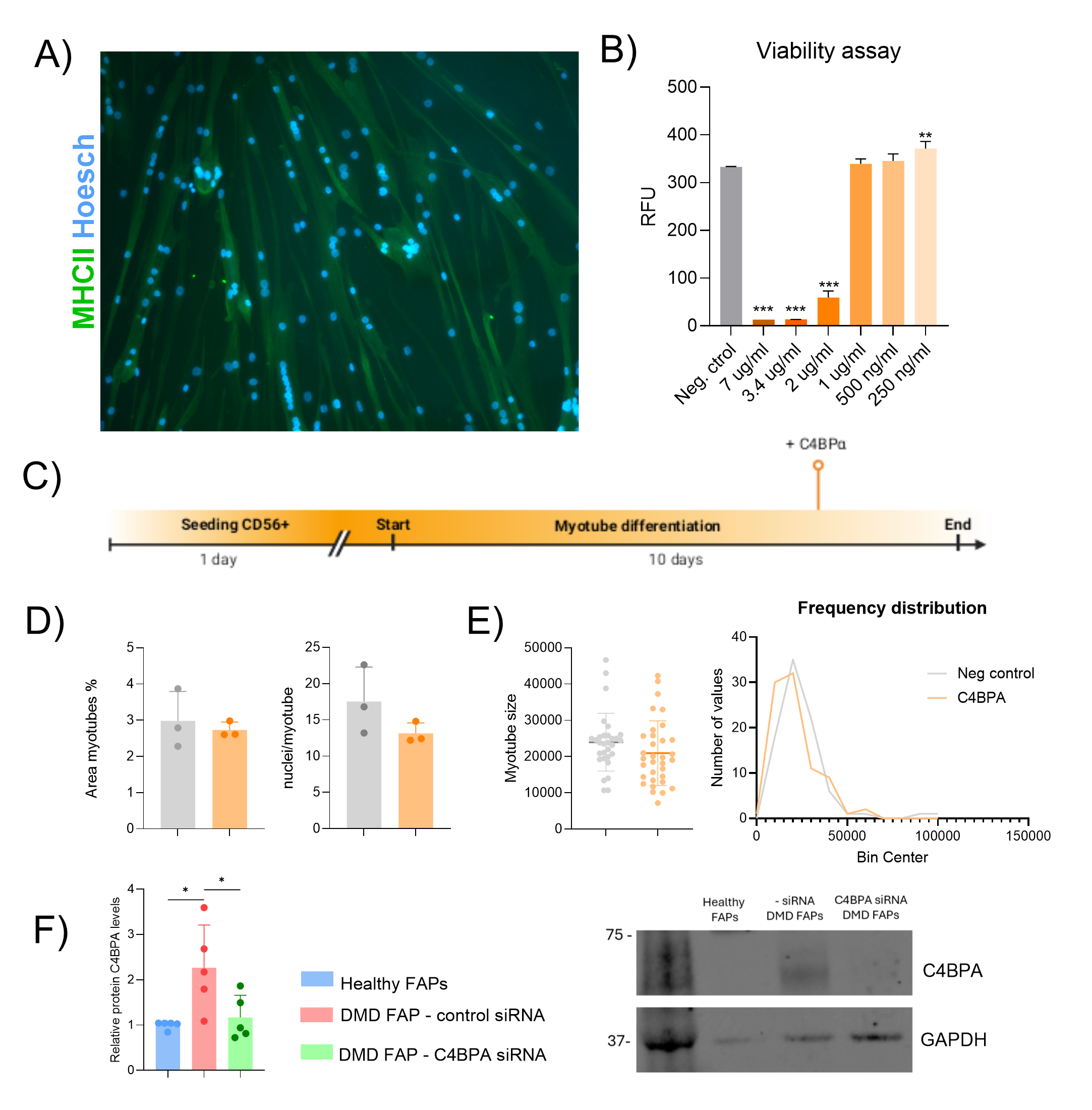

Supplement: Supplementary file 2 — Supplemental Figure 1 [file 41419_2026_8588_MOESM2_ESM.tif]
