# Supplementary material for: Impact of C4BPA on Muscle progenitor cell differentiation: insights for Duchenne muscular dystrophy treatment
Source: Cell Death Dis. 2026 Mar 18;17(1):313. doi: 10.1038/s41419-026-08588-2 (PMC13039365; doi:10.1038/s41419-026-08588-2)

Full western blot images

Muscle tissue WB corresponding to Figure 3E)

GAPDH C4BPA


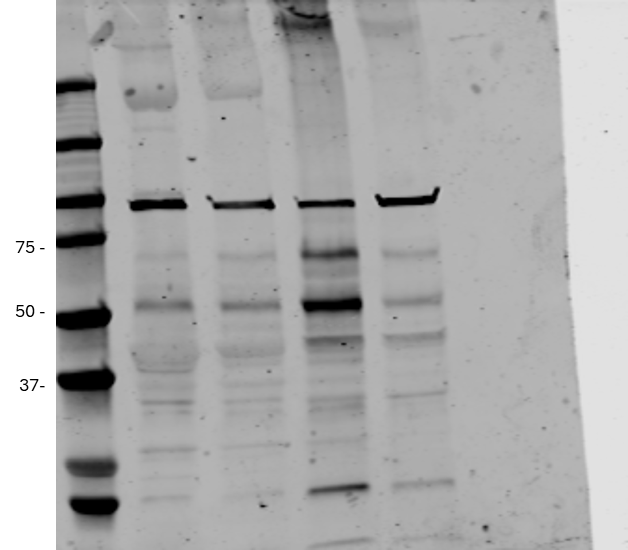

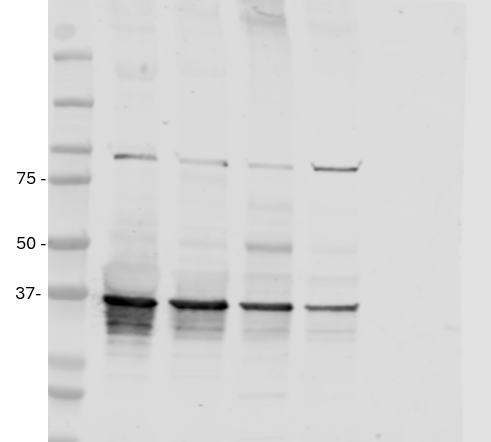


37-

50 -

75 -

Cell lines corresponding to Figure 3E)

DMD cell line - C4BPA + GAPDH


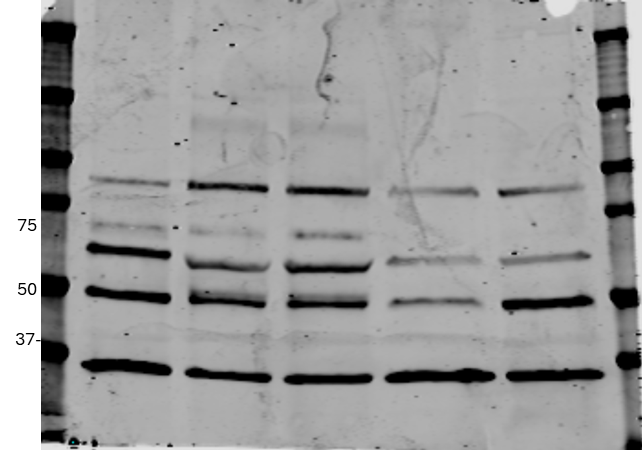


Healthy control - C4BPA + GAPDH


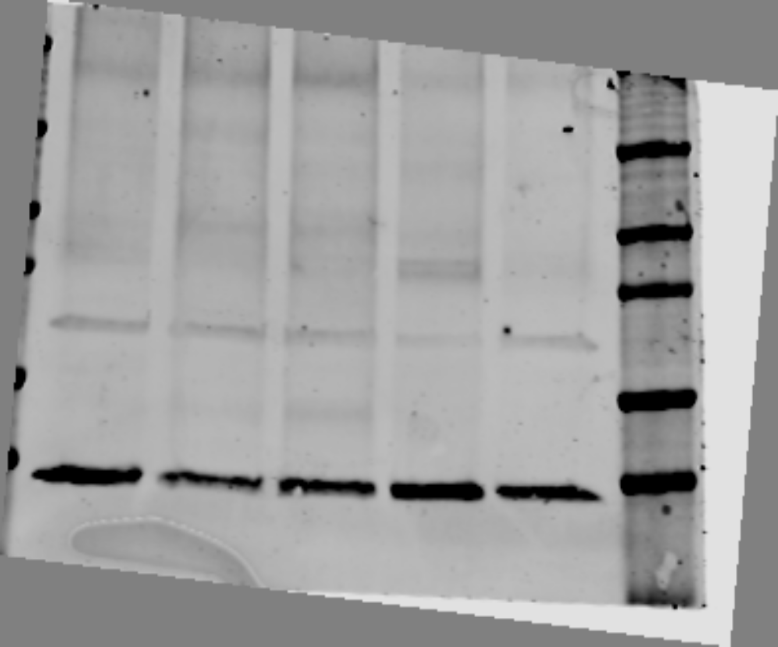


37-

50 -

75 -

siRNA WB – Supplemental 1F

C4BPA + GAPDH


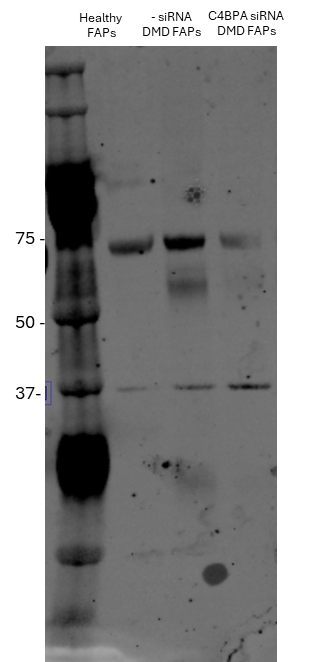

Supplement: Supplementary file 3 — Full WB [file 41419_2026_8588_MOESM3_ESM.docx]
